# Speckle tracking echocardiography for evaluation of myocardial functions before and after mitral valvuloplasty in dogs

**DOI:** 10.3389/fvets.2024.1463889

**Published:** 2024-10-28

**Authors:** Sho Fukuzumi, Ahmed S. Mandour, Ahmed Farag, Tomohiko Yoshida, Akitsugu Nishiura, Hideki Yotsuida, Yota Yaginuma, Katsuhiro Matsuura, Ryou Tanaka

**Affiliations:** ^1^VCA Japan Dolphin Animal Hospital Urawamisono, Saitama, Japan; ^2^Department of Veterinary Teaching Hospital, Faculty of Agriculture, Tokyo University of Agriculture and Technology, Tokyo, Japan; ^3^Department of Animal Medicine (Internal Medicine), Faculty of Veterinary Medicine, Suez Canal University, Ismailia, Egypt; ^4^Department of Small Animal Medical Center, Obihiro University of Agriculture and Veterinary Medicine, Obihiro, Japan; ^5^Ashiya Limone Animal Hospital, Ashiya, Japan; ^6^Department of Clinical Engineering, National Hospital Organization Osaka National Hospital, Osaka, Japan; ^7^VCA Japan Shiraishi Animal Hospital, Saitama, Japan; ^8^Department of Small Animal Clinical Sciences, College of Veterinary Medicine University of Florida, Gainesville, FL, United States

**Keywords:** mitral valve repair, 2D-STE, twist, GLS, GCS, GRS, ventricular interaction

## Abstract

**Background:**

Myxomatous mitral valve disease (MMVD) is the most common acquired heart disease in dogs. Mitral valvuloplasty (MVP) addresses regurgitation, but the pre- and postoperative changes in myocardial function remain uncertain.

**Objectives:**

This study evaluated myocardial motion before and after MVP using two-dimensional speckle-tracking echocardiography (2D-STE).

**Animals:**

Eight client-owned dogs undergoing MVP for MMVD.

**Methods:**

Myocardial deformation was assessed by 2D-STE before surgery and at 1- and 3-months post-surgery. Measurements included left ventricular global longitudinal strain (GLS), global circumferential strain (GCS), global radial strain (GRS), cardiac twist, and right ventricular free wall GLS (RVFW-GLS).

**Results:**

Postoperative decreases were observed in left ventricular internal dimensions, left atrial size, and early diastolic myocardial velocity, with an increase in peak late diastolic velocity. LV-GLS decreased at 1 month (−14.4%) and 3 months (−16.3%) compared to preoperative values (−24.4%) (*p* = 0.0078, *p* = 0.015). GCS decreased at 1 month (−12.9%) and 3 months (−14.8%) compared to preoperative values (−21.7%) (*p* = 0.0078). GRS decreased at 1 month (27.7%) and 3 months (32.0%) compared to preoperative values (67.7%) (*p* = 0.0078). No significant changes were observed in RVFW-GLS. Peak systolic twist increased at 3 months (9.1° vs. 4.9°, *p* = 0.039). Peak systolic apical rotation showed an upward trend at 3 months (*p* = 0.109). Left ventricular twist was mildly affected by LVIDd, LVIDDN, and sphericity index (*R*^2^ = 0.187, *p* = 0.034; *R*^2^ = 0.33, *p* = 0.0029; *R*^2^ = 0.22, *p* = 0.019).

**Conclusions and clinical importance:**

Postoperative myocardial motion approached reference values, indicating significant improvement, particularly in left ventricular twisting motion. These findings highlight the positive impact of surgery on cardiac function in dogs with MMVD.

## 1 Introduction

Myxomatous mitral valve degeneration (MMVD) stands as a primary factor necessitating valve repair or replacement as it leads to mitral regurgitation, cardiac enlargement, systolic dysfunction, and eventual heart failure (1). Recent insights have underscored the potential of mitral valvuloplasty (MVP) to facilitate full recuperation, MVP emerges as a promising therapeutic avenue for MMVD, fostering reverse remodeling and restoring the dilated dimensions of the left atrium and left ventricle to their physiological state (2, 3).

Left ventricular fractional shortening (FS) is commonly used to assess left ventricular systolic function in veterinary medicine. Preoperative FS often increases due to improved preload. However, post-surgery, FS tends to normalize, although some cases show persistently low FS values despite the absence of heart failure signs (4), indicating that conventional echocardiography alone may not provide a precise assessment of postoperative cardiac function.

Two-dimensional speckle-tracking echocardiography (2D-STE) is an advanced imaging technique that allows for comprehensive evaluation of myocardial function through deformation imaging. It provides valuable insights into both global and regional myocardial function, as well as the ability to quantify cardiac rotation and synchronicity parameters not accessible with conventional echocardiography (5).

Resolution of regurgitation and congestion before and after mitral valve surgery has been observed (4). However, it’s unclear if this mitigated dysfunction translates into postoperative improvement or simply slows progression. Precise evaluation of surgery’s impact on cardiac function is crucial to substantiate its efficacy.

Speckle-tracking strain analysis has demonstrated precise reflection of LV myocardial function with angle-independent assessment (6). Regarding Global longitudinal strain (GLS), earlier studies have indicated a decrease in GLS in both ventricles post-surgery (7). However, in veterinary medicine, preoperative left ventricular GLS was reported to remain unchanged depending on the disease stage (8), and alterations in GLS following surgery have not yet been documented. Global circumferential strain (GCS) and Global radial strain (GRS) have been reported to increase with enlargement of the left ventricular chamber (8). However, the postoperative course of these parameters remains unclear. The subepicardial and subendocardial fibers are warped around the LV into left- and right-handed helix causing clockwise and counterclockwise rotation of the base and the apex. The left ventricular rotational deformation includes systolic twist and diastolic untwist, resembling the wringing and unwringing of a towel, can be evaluated by 2D-STE. Twist has been noted to deteriorate in severe MMVD (9), where the LV twist variables were decreased with increasing systolic LV internal diameter (10), suggesting that twisting and untwisting may improve following MVP. The postoperative changes of twist in dogs undergoing MVP remains unexplored.

This study aims to clarify improvements in GLS and twist of both ventricles after MVP in MMVD cases. GLS is a sensitive indicator of longitudinal myocardial fiber function, and twist reflects the torsional mechanics of the heart, both of which are critical for effective cardiac output. Monitoring these parameters post-surgery provides valuable insights into the heart’s recovery and reverse remodeling process, which may not be fully captured by conventional echocardiography alone. Understanding these changes can help optimize post-surgical management and further refine surgical techniques. By analyzing GLS and twist changes post-surgery, it seeks to address limitations in evaluating postoperative cardiac function solely through conventional echocardiography, emphasizing 2D-STE.

## 2 Materials and methods

### 2.1 Animals

Table 1 describes the signalments of the patients included in the study. Eight client-owned dogs undergoing mitral valvuloplasty (MVP) exclusively for MMVD between January 2022 and April 2023 at Dolphin Animal Hospital Urawamisono were recruited for this study. Prior to enrollment, informed consent was obtained from the respective dog owners. Diagnosis of MMVD was established through conventional cardiac echocardiography, and dogs were categorized according to the guidelines set by the American College of Veterinary Internal Medicine (ACVIM). Among the eight dogs, three were classified as Stage B2, and five as Stage D. Each dog received treatment tailored to their specific stage. Some Stage B2 cases were already receiving diuretic medication prior to MVP. Baseline measurements were recorded within 1 month before the scheduled surgery date. Post-MVP, the dogs underwent a two-week course of pimobendan (Pimobeheart, Kyoritsu Seiyaku Corporation, Tokyo, Japan), Potassium Clavulanate Amoxicillin Hydrate (Augmentin, GlaxoSmithKline K.K., Tokyo, Japan), Orbifloxacin (Victas^®^, Bussan Animal Health Co., Ltd., Osaka, Japan), and rivaroxaban (Rivaroxaban, Bayer Yakuhin, Ltd., Osaka, Japan) administration. Following this period, all cases ceased the use of inotropic agents and antibiotics, while rivaroxaban continued for 3 months postoperatively. In one instance, amlodipine besilate (Norvasc Tablets, Viatris Pharmaceutical Co., Ltd., Tokyo, Japan) administration resumed 2 weeks after surgery, while in another case, benazepril hydrochloride (FORTECOL^®^, Elanco Japan Co., Ltd., Tokyo, Japan) and amlodipine were maintained due to chronic kidney disease.

**Table 1:** Demographic data of the canine patients undergoing MVP and STE analysis.

No.	Breed	Gender	Age (year)	BW (kg)	ACVIM stage	Cardiac medication (mg/kg)	Day of discharge after surgery	STE data
1	Pomeranian	FS	10	4.15	D	P 0.35 q8h	6	Yes
						B 0.35 q12h		
						A 0.10 q12h		
						T 0.14 q12h		
						H 0.52 q12h		
						T 0.42 q12h		
						I 0.52 q12h		
						G 0.53 mEq/kg q12h		
2	CKCS	FS	12	7.92	D	P 0.67 q12h	5	Yes
						B 0.67 q12h		
						A 0.17 q12h		
						T 0.15 q12h		
						H 1.27 q12h		
						G 0.88 mEq/kg q24h		
3	Papillion	FS	8	4.3	D	P 0.58 q8h	6	Yes
						B 0.29 q12h		
						T 0.03 q12h		
						To 0.195 q12h		
						S 0.22 q12h		
4	Pomeranian	MN	12	4.76	D	P 0.58 q12h	6	Yes
						Al 2.9 q12h		
						A 0.15 q12h		
						T 0.15 q12h		
						H 0.45 q12h		
						Sp 2.9 q12h		
5	Chihuahua	FS	9	3.9	B2	P 0.48 q12h	4	Yes
						A 0.12 q12h		
						F 0.32 q12h		
6	Chihuahua	FS	12	2.54	B2	P 0.49 q12h	5	Yes
						B 0.49 q12h		
						A 0.1 q12h		
						T 0.08 q24h		
7	Chihuahua	MN	9	6.6	B2	P 0.38 q12h	5	Yes
						A 0.09 q12h		
8	Pomeranian	FS	10	2.98	D	P 0.38 q12h	4	Yes
						A 0.13 q12h		
						T 0.1 q12h		
						H 0.8 q12h		

CKCS, Cavalier King Charles Spaniel; MN, male neutered; FS, female spayed; P, pimobendan; B, benazepril; Al, alacepril; A, amlodipine; F, frosemide; T, torsemide; H, hydrochlorothiazide; Sp, spironolactone; To, tolvaptan; I, isosorbide dinitrate; G, gluconate potassium; S, sildenafil.

In two out of eight cases, medications impacting hemodynamics were introduced 2 weeks post-surgery (in case 3: amlodipine and benazepril, in case 4: amlodipine). Nonetheless, the administration of pimobendan and diuretics, pivotal in managing MMVD, was ceased in all cases 2 weeks following MVP.

### 2.2 Mitral valvuloplasty

Mitral valvuloplasty was conducted following established procedures as previously described (2, 11). In summary, an incision was made in the left fifth intercostal chest, and the pericardial tent was deployed. A cannula containing myocardial protection fluid was then inserted into the aorta. Cardiac arrest was induced using myocardial protection after aortic blockade with an extracorporeal circulatory system. Subsequently, access to the mitral valve apparatus was gained through a left atrial incision. Reconstruction of the mitral annulus was performed, followed by closure of the left atrium. Upon completion, the cannula was removed, and the patient was gradually weaned from extracorporeal circulation following the release of cardiac arrest.

### 2.3 Echocardiography

#### 2.3.1 Conventional echocardiography

Echocardiographic assessments were conducted utilizing a Hitachi Arietta 60 or Hitachi Arietta 750 ultrasound system, both manufactured by Hitachi Aloka Medical in Tokyo, Japan, equipped with a 2–9 MHz probe. Standard conventional echocardiography was performed from both right and left sides (12), encompassing ordinary long-axis and short-axis views for each dog at baseline (preoperative) and two time points postoperative (at 1 and 3 months).

The following conventional echocardiographic indices were measured: left ventricular internal dimension in diastole (LVIDd), LVIDd normalized to body weight, using an allometric approach (LVIDDN), fractional shortening, peak mitral E wave velocity, peak mitral A wave velocity, the E-to-A ratio, and mitral regurgitation (MR) velocity. Additionally, the ratio of the left atrial dimension to the aortic annulus dimension (LA/Ao) was measured at the basal level using two-dimensional echocardiography. Tissue Doppler imaging was utilized to measure myocardial velocity during early diastole (E′) at the lateral annulus and septal annulus, as well as the tricuspidal velocity during systole (S′) at the lateral annulus. LV sphericity index (LVSI) was calculated as the ratio of the long axis to the short axis of the left ventricle (13).

#### 2.3.2 Two-dimensional speckle tracking echocardiography

Left ventricular (LV) twist was assessed utilizing basal- and apical-level images from the right parasternal short-axis view. Left ventricular global longitudinal strain (LV-GLS) was derived from the apical four-chamber section. Global circumferential strain (GCS) and global radial strain (GRS) were obtained at the papillary muscle level images from the right parasternal short-axis view (14). Analysis of right ventricular (RV) free wall global longitudinal strain (RVFW-GLS) was conducted using RV-focused view. Digital cine-loops capturing three consecutive heart cycles, with a frame rate ranging from 72 to 86 frames per second, were acquired from all dogs and stored as DICOM files for subsequent processing on echo machine. Data from three dogs (Nos. 6, 7, 8 on Table 1), where accurate evaluation of RVFW-GLS was hindered due to issues with the acquired images, were excluded from analysis.

### 2.4 Statistical analysis

The data were presented as median and interquartile range. Statistical analyses were conducted using commercial statistical software (Prism 10.0v; GraphPad Software Inc., San Diego, CA, United States). Preoperative and postoperative 1-month, as well as preoperative and postoperative 3-month data obtained from conventional echocardiographic examination and 2D-STE analysis, were assessed using the Wilcoxon signed-rank test, respectively. A *p*-value less than 0.05 was considered to indicate statistical significance for the Gaussian approximation of nonparametric data. Nonparametric data are presented as median and range. Additionally, the relationship between the data obtained from conventional echocardiographic examination and 2D-STE analysis or between 2D-STE analysis was evaluated using simple linear regression analysis. A significance level of *p* < 0.05 was considered statistically significant.

## 3 Results

### 3.1 Echocardiographic measurement

#### 3.1.1 Conventional echocardiography

**Figure 1:**
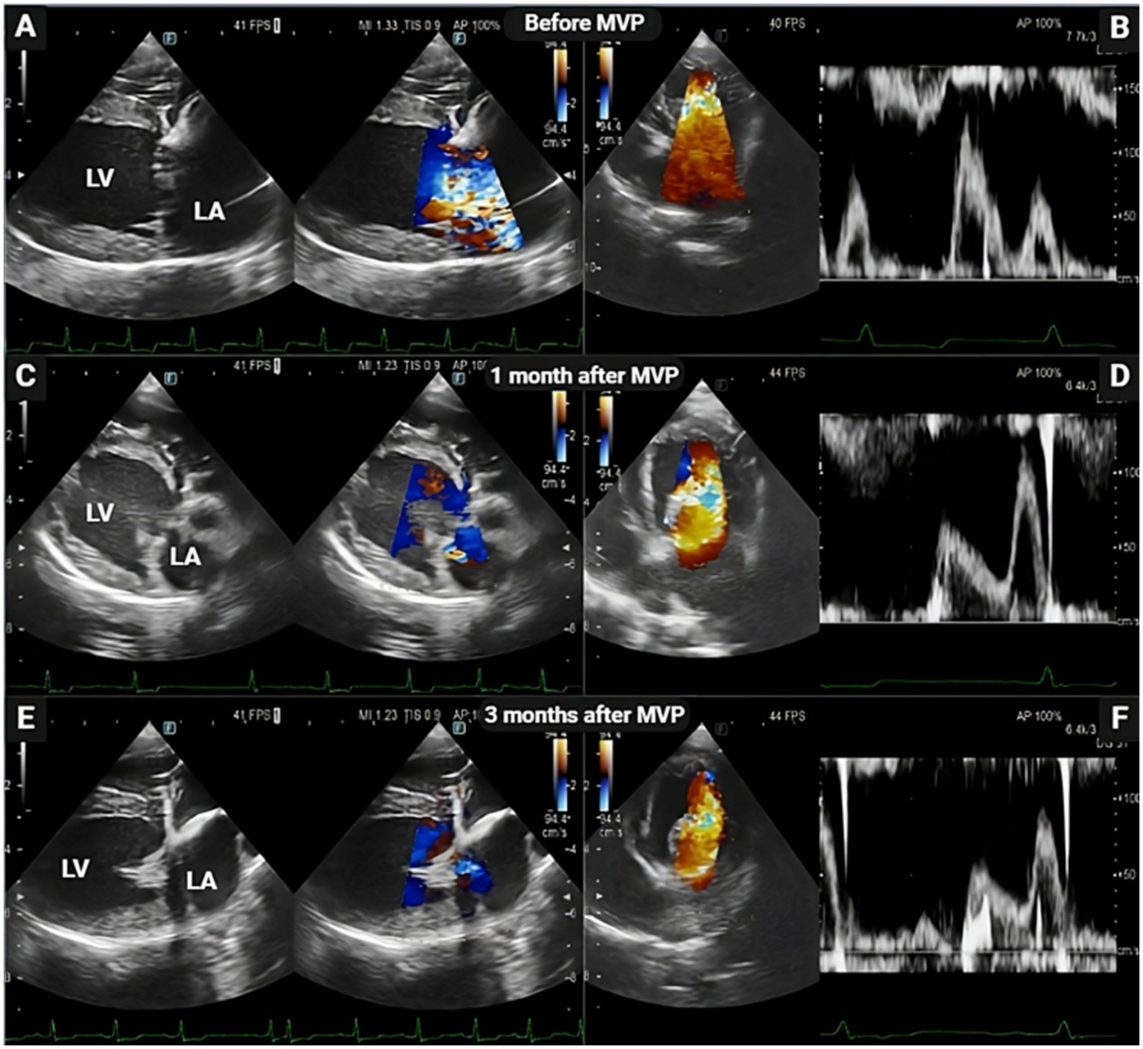
Representative echocardiogram images before, 1 month, and 3 months after mitral valvuloplasty (MVP) in dog patients. Two-dimensional echocardiography at the long-axis LV inflow view demonstrating the thickening of the mitral valve, enlarged left ventricle and left atrium chambers with mitral valve regurgitation (A) and elevated mitral inflow velocity (B) before MVP. Following mitral MVP, there was significant restoration in cardiac functional parameters, characterized by reduced left ventricular and atrium lumen size (C,E) and reduced early mitral inflow velocities (D,F). LA, left atrium; LV, left ventricle.

The results of the conventional echocardiographic examination are presented in Table 2. Heart rate (HR), LVIDd, LVIDDN, LA/Ao, FS, E′ sep, E′ lat exhibited significant decreases at both 1 month and 3 months post-surgery compared to preoperative values, while mitral A velocity significantly increased at 3 months post-surgery. Additionally, LVSI significantly increased at both 1 month and 3 months after MVP. Furthermore, E/E′ sep significantly decreased at 1 month after surgery compared to preoperative values. Body weight (BW) showed a significant decrease at 1 months after surgery compared to preoperative values (Figure 1).

**Table 2:** Conventional echocardiography results for 8 dogs preoperatively, 1 month, and 3 months postoperatively.

Parameters	Unit	Baseline	Postoperative	
			1 month	3 months
BW	kg	4.23 (3.67–5.22)	3.70 (3.29–4.83)*	3.83 (3.28–4.93)
HR	beats/min	132 (127–167)	120 (114–130)*	111 (102–129)*
LVIDd	mm	29.5 (23.6–36.2)	18.8 (17.9–20.8)**	21.2 (17.2–23.8)**
LVIDDN		2.03 (1.70–2.33)	1.30 (1.26–1.39)**	1.35 (1.15–1.60)**
LA/Ao		2.00 (1.72–2.44)	1.34 (1.17–1.45)*	1.36 (1.16–1.49)*
FS%	%	56.2 (50.1–60.6)	30.5 (28.6–33.0)*	35.3 (28.7–44.9)*
Mitral E velocity	cm/s	112.9 (81.8–127.4)	75.7 (70.4–85.6)	74.7 (68.6–87.6)
Mitral A velocity	cm/s	84.7 (81.0–92.1)	104.1 (93.3–111.6)	106.9 (100.3–109.2)*
E/A		1.08 (0.96–1.59)	0.79 (0.61–0.92)	0.68 (0.63–0.84)*
E′ sep	cm/s	7.9 (6.4–8.7)	3.9 (2.9–4.7)**	4.4 (3.6–4.9)**
E′ lat	cm/s	8.8 (7.5–9.1)	4.4 (3.6–4.7)*	4.1 (3.8–4.9)*
E/E′ sep		14.8 (11.8–18.9)	19.4 (17.7–28.0)*	17.9 (16.5–19.2)
E/E′ lat		12.2 (10.3–15.6)	19.0 (15.3–19.5)	16.6 (13.6–19.5)
MR velocity	m/s	5.7 (5.5–6.0)	Trivial	Trivial
LV sphericity index		1.25 (1.01–1.32)	1.34 (1.30–1.65)**	1.46 (1.32–1.53)**
RV S′	cm/s	14.9 (13.8–18.1)	10.3 (8.4–11.2)	10.2 (10.0–11.7)

Data are expressed as medians (interquartile ranges). Asterisk (*) is used to compare the significance between values of preoperative and postoperative observations (*, ** indicates *p* < 0.05, 0.01, respectively). LVIDd, left ventricular internal dimension in diastole; LVIDDN, normalized left ventricular internal dimension in diastole; LA/Ao, the ratio of the left atrial dimension to the aortic annulus dimension; FS, fractional shortening; E velocity, early diastolic mitral inflow velocity; A velocity, late diastolic mitral inflow velocity; E/A, the ratio of peak velocity of early diastolic transmitral flow to peak velocity of late diastolic transmitral flow; E′, early diastolic wave signal as measured by tissue Doppler imaging; sep, mitral annulus at the septal wall; lat, mitral annulus at the left ventricular lateral wall; MR velocity, mitral regurgitation velocity; LV sphericity index, left ventricular sphericity index; RV S′, tricuspidal systolic wave signal as measured by tissue Doppler imaging.

#### 3.1.2 Two-dimensional speckle tracking echocardiography

**Figure 2:**
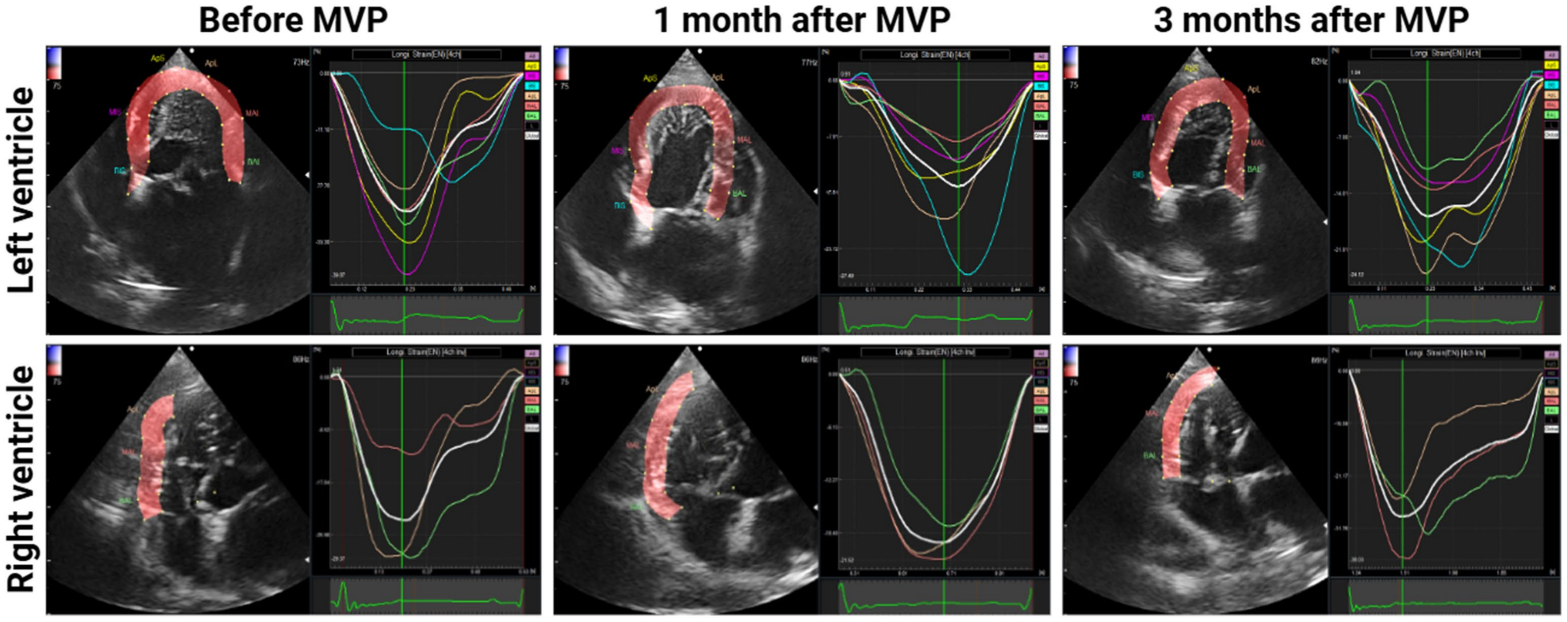
Longitudinal strains of the left and right ventricles before, at 1 month, and at 3 months After MVP using two-dimensional speckle tracking echocardiography. A central line demonstrated the downward peak strain was captured.

**Figure 3:**
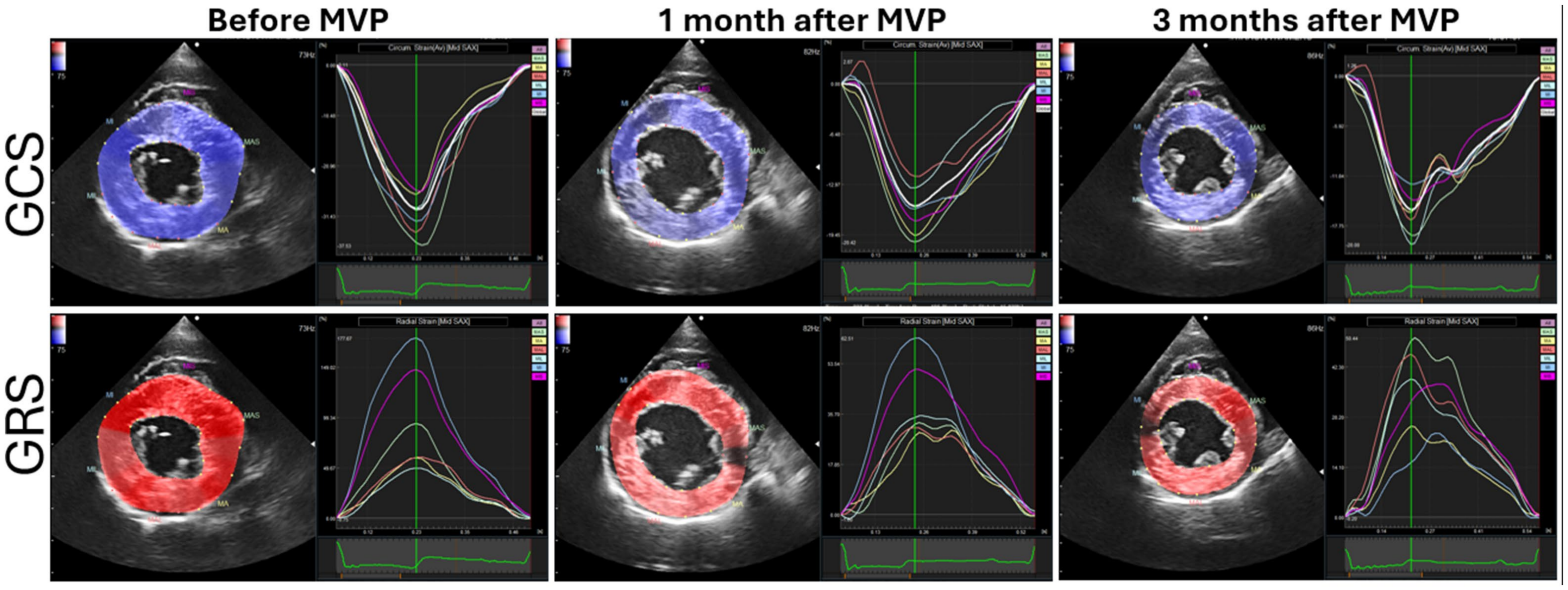
Global circumferential strain (GCS) and global radial strain (GRS) before, at 1 month, and at 3 months after mitral valvuloplasty (MVP) using 2D-STE.

**Figure 4:**
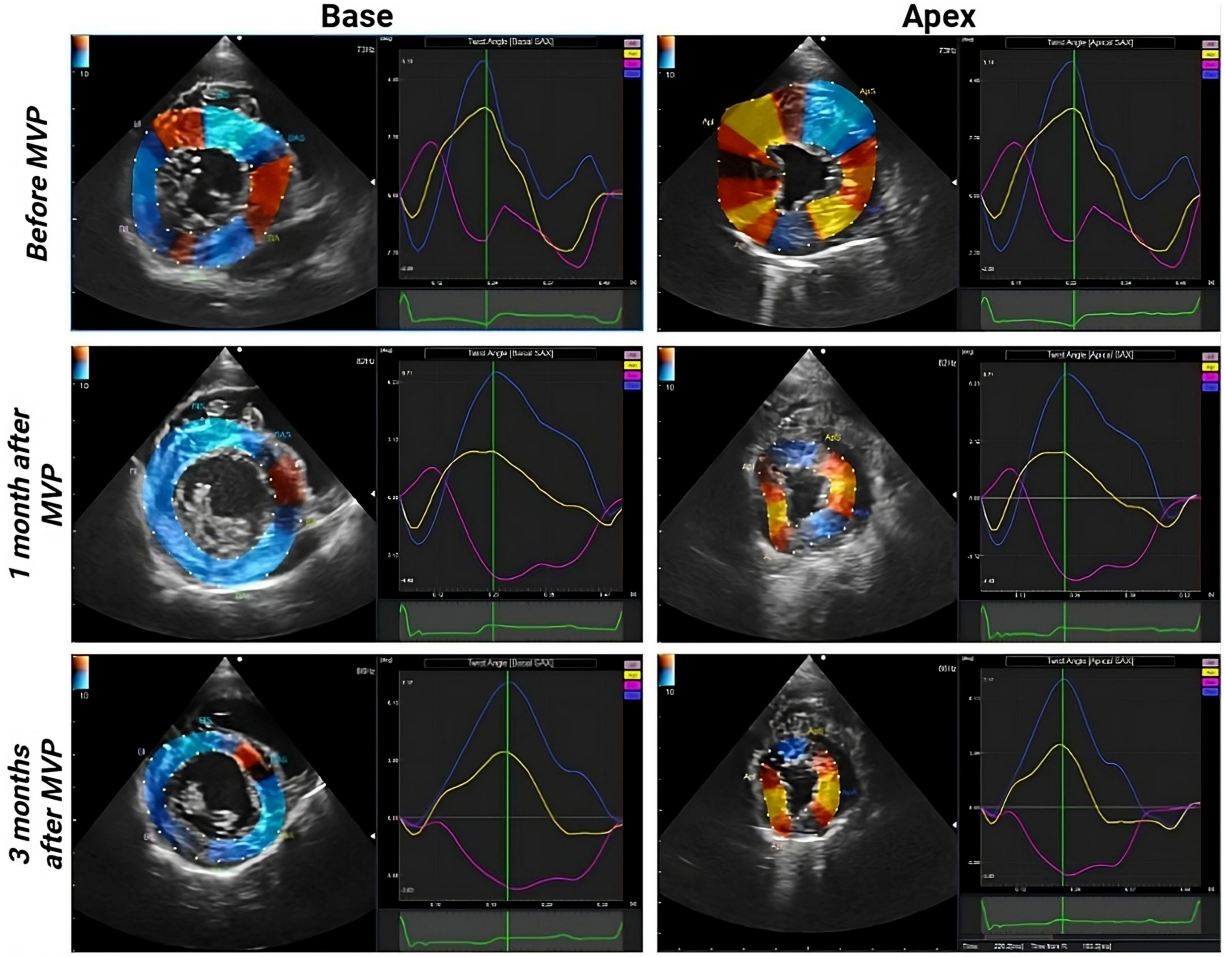
Left ventricular (LV) twist before, at 1 month, and at 3 months after MVP. Blue curve: LV twist, yellow curve: apex rotation, pink curve: base rotation.

Figure 2 depict the longitudinal strains of the left and right ventricles utilizing 2D-STE, while Figure 3 showcases the global circumferential strain (GCS) and global radial strain (GRS) before, at 1 month, and at 3 months post MVP using two-dimensional speckle tracking echocardiography. Similarly, Figure 4 illustrates the LV twist at various time points before and after MVP.

**Figure 5:**
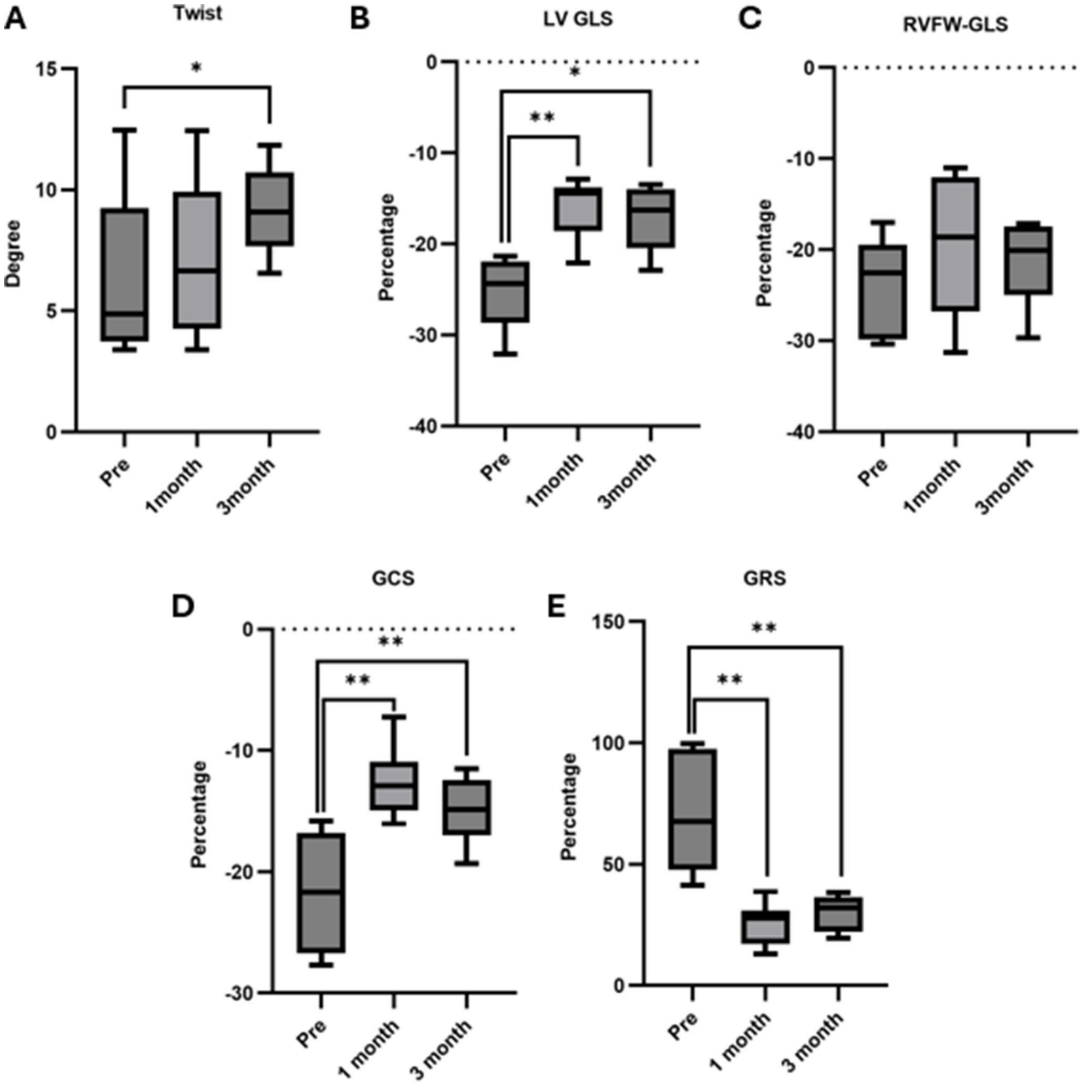
Twist, LV GLS, RVFW GLS Analysis via 2D-STE. (A) Twist: no significant changes were noted between preoperative and 1-month postoperative assessments, but a notable increase was observed at 3 months postoperatively compared to preoperative (*p* = 0.039). (B) LV-GLS: there was a significant decrease at 1 month (*p* = 0.0078) and 3 months (*p* = 0.0156) post-surgery compared to preoperative. (C) RVFW-GLS: no significant changes were observed at 1 month and 3 months post-surgery compared to preoperative. (D) GCS: there was a significant decrease at 1 month (*p* = 0.0078) and 3 months (*p* = 0.0078) post-surgery compared to preoperative. (E) GRS: there was a significant decrease at 1 month (*p* = 0.0078) and 3 months (*p* = 0.0078) post-surgery compared to preoperative. Data are presented as medians with interquartile ranges. An asterisk (*) indicates significance in the comparison between preoperative and postoperative values (*indicates *p* < 0.05, **indicates *p* < 0.01).

LV-GLS, GCS, and GRS exhibited a significant decrease at both 1 month (*p* = 0.0078, 0.0078, 0.0078, respectively) and 3 months (*p* = 0.0156, 0.0078, 0.0078, respectively) post-surgery compared to preoperative values. Notably, no significant changes were observed in RVFW-GLS at any time point compared to the preoperative values. The results of the offline workstation analysis for all eight cases are presented in Table 3. Reference values were obtained from past reports (8, 9, 15, 16). In the 3-month postoperative group, peak systolic twist demonstrated a significant increase compared to the preoperative group (*p* = 0.039) (Figure 5). Peak systolic apical rotation increased at 3 months postoperatively compared to the preoperative values, although statistical significance was not reached (*p* = 0.1094).

**Table 3:** Two-dimensional speckle-tracking echocardiography results for 8 dogs preoperatively and at 1 month and 3 months postoperatively.

Parameters	Unit	Preoperative	Postoperative		Reference value
			1 month	3 months	
Peak systolic twist	°	4.9 (4.2–7.8)	6.6 (4.2–8.8)	9.1 (7.8–10.1)*	15.3 (12.7–18.7)
Peak systolic twist rate	°/s	86.1 (61.0–115.5)	89.2 (58.5–111.9)	91.7 (88.2–105.5)	176.3 (159.8–231.6)
Peak systolic apical rotation	°	2.7 (2.3–6.0)	3.3 (2.3–5.0)	6.1 (5.1–6.7)	10.7 (9.0–13.8)
Peak systolic basal rotation	°	−2.9 [(−4.0) to (−1.5)]	−1.9 [(−4.0) to (−1.3)]	−3.5 [(−3.9) to (−2.6)]	−7.0 [(−8.4) to (−4.1)]
LV-GLS	%	−24.4 [(−26.6) to (−22.1)]	−14.4 [(−16.8) to (−13.8)]**	−16.3 [(−18.6) to (−14.3)]*	−19 [(−23) to (−14)]
GCS	%	−21.7 [(−26.1) to (−17.4)]	−12.9 [(−14.4) to (−11.1)]**	−14.8 [(−16.2) to (−13.7)]**	−15.44 ± 1.50
GRS	%	67.7 (49.0–95.6)	27.7 (23.3–30.8)**	32.0 (23.2–36.5)**	31.96 ± 7.12
RVFW-GLS^a^	%	−22.5 [(−29.3) to (−21.9)]	−18.6 [(−22.3) to (−13.0)]	−18.9 [(−20.3) to (−17.3)]	−19.0 ± 3.1

Data are expressed as medians (interquartile ranges). Asterisk (*) is used to compare the significance between values of preoperative and postoperative observations (*indicates *p* < 0.05, **indicates *p* < 0.01).

a Values in 5 cases were shown due to data not yet obtained.

**Figure 6:**
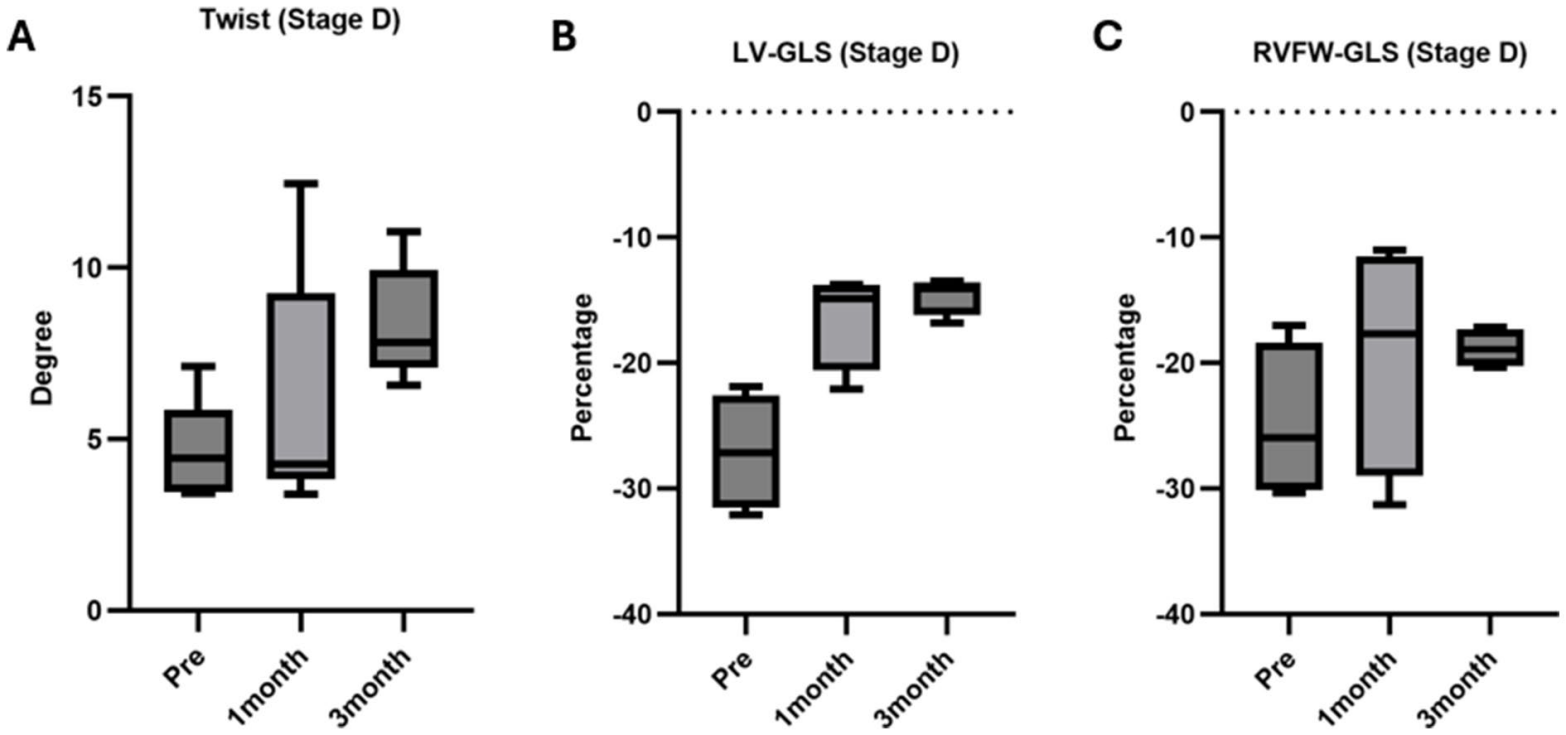
Twist, LV GLS, RVFW GLS analysis via 2D-STE in ACVIM Stage D cases. (A) Twist: no significant differences were noted at 1 month (*p* = 0.3125) and 3 months (*p* = 0.0625) post-surgery compared to preoperative values. (B) LV GLS: no significant differences were observed at 1 month (*p* = 0.1250) and 3 months (*p* = 0.1250) post-surgery compared to preoperative values. (C) RVFW GLS: no significant differences were observed at 1 month (*p* = 0.3750) and 3 months (*p* = 0.2500) post-surgery compared to preoperative values.

The analysis results for cases classified as MMVD Stage D are summarized in Table 4. When comparing preoperative and postoperative 1-month data, no significant differences were observed in any of the measured 2D-STE parameters. Similarly, when comparing preoperative and three-month postoperative values in Stage D, a trend change was noted in peak systolic twist (*p* = 0.0625), peak systolic apical rotation (*p* = 0.0625), LV-GLS (*p* = 0.1250), GCS (*p* = 0.0625), GRS (*p* = 0.0625), and RVFW-GLS (*p* = 0.2500) (Figure 6).

**Table 4:** Two-dimensional speckle-tracking echocardiography results for 5 dogs (Stage D) preoperatively, 1 month, and 3 months postoperatively.

Parameters	Unit	Preoperative	Postoperative		Reference value
			1 month	3 months	
Peak systolic twist	°	4.4 (3.5–4.6)	4.3 (4.2–6.1)	7.8 (7.6–8.8)	15.3 (12.7–18.7)
Peak systolic twist rate	°/s	80.5 (61.0–91.6)	62.3 (47.1–88.8)	88.6 (86.9–93.7)	176.3 (159.8–231.6)
Peak systolic apical rotation	°	2.5 (1.8–2.6)	2.4 (2.4–4.5)	5.5 (3.7–6.0)	10.7 (9.0–13.8)
Peak systolic basal rotation	°	−1.9 [(−3.8) to (−1.8)]	−1.4 [(−1.9) to (−1.2)]	−3.3 [(−3.9) to (−2.8)]	−7.0 [(−8.4) to (−4.1)]
LV-GLS	%	−24.6 [(−29.7) to (−21.9)]	−14.7 [(−16.0) to (−13.8)]	−14.4 [(−16.8) to (−13.8)]	−19 [(−23) to (−14)]
GCS	%	−22.0 [(−27.0) to (−21.4)]	−11.9 [(−15.2) to (−11.2)]	−15.3 [(−15.8) to (−14.4)]	−15.44 ± 1.50
GRS	%	94.7 (72.3–98.5)	26.4 (14.1–31.0)	30.4 (21.7–33.5)	31.96 ± 7.12
RVFW-GLS^a^	%	−25.9 [(−29.6) to (−21.2)]	−17.7 [(−24.5) to (−12.5)]	−18.9 [(−20.1) to (−17.6)]	−19.0 ± 3.1

Data are expressed as medians (interquartile ranges).

a Values in 4 cases were shown due to data not yet obtained.

### 3.2 Effect of conventional echocardiographic parameters on twist

**Figure 7:**
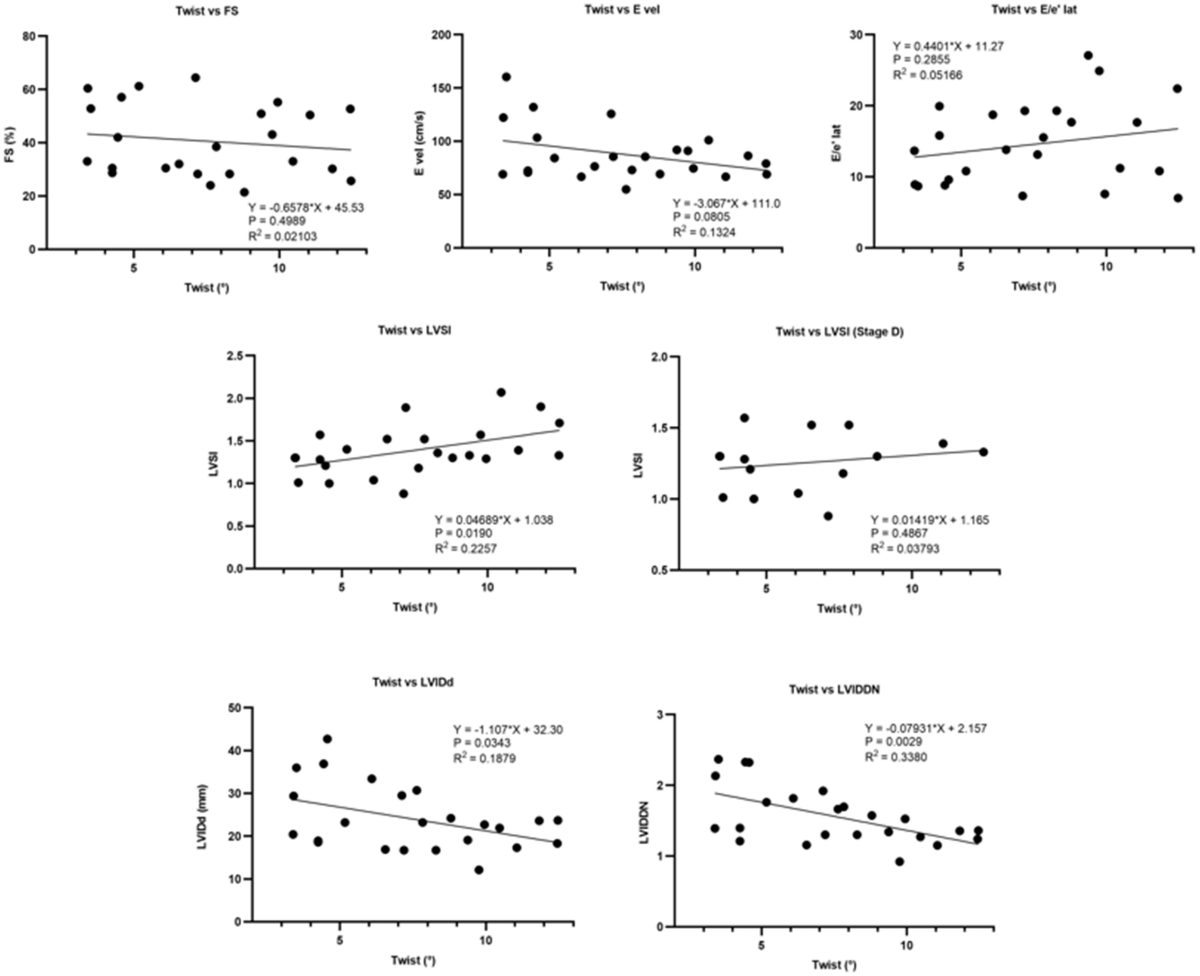
Linear regression analysis between indices of contractility (FS), congestion (E velocity), diastolic function (E/e′), left ventricular sphericity index (LVSI), left ventricular internal diameter in diastole (LVIDd), left ventricular internal diameter in diastole normalized (LVIDDN), and twist, FS, E velocity, and E/e′ lateral did not exhibit a significant regression with twist. In contrast, LVIDd, LVIDDN, and LVSI showed a significant impact on twist.

The coefficient of determination (*R*^2^) obtained from linear regression analysis between indices of contractility (FS), congestion (E velocity), diastolic function (E/e′), left ventricular sphericity index (LVSI), LVIDd, LVIDDN, and twist was illustrated in Figure 7. FS, E velocity, and E/e′ lateral did not exhibit a significant effect on twist. In contrast, LVIDd and LVSI showed a mild significant impact on twist (*p* = 0.0343, and 0.0190, respectively).

**Figure 8:**
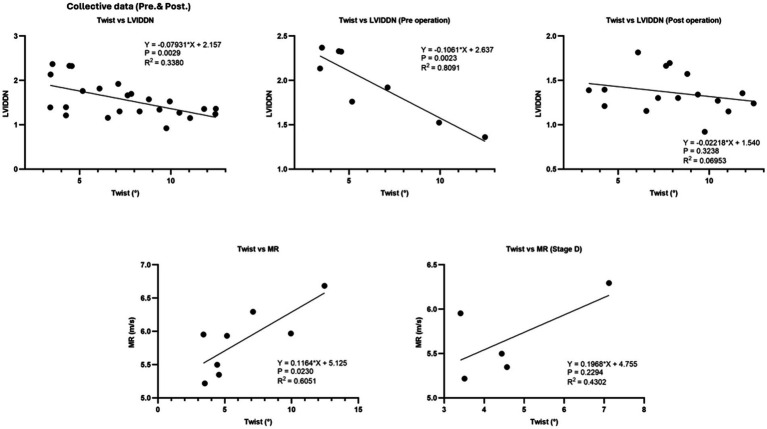
The collective data demonstrated a mild significant effect of LVIDDN on twist. However, the effect of LVIDDN on twist was notably stronger when considering only the preoperative data. Additionally, the degree of cardiac twisting was positively influenced by the regurgitation velocity preoperatively.

The collective data showed mild significant effect of LVIDDN on twist (*R*^2^ = 0.3380, *p* = 0.00229). However, the effect of LVIDDN on twist was stronger when considering only the preoperative data (*R*^2^ = 0.809, *p* = 0.0023). Preoperatively, the degree of cardiac twisting was positively affected by the MR velocity (*R*^2^ = 0.605, *p* = 0.023) (Figure 8).

### 3.3 Effect of GCS and GRS on conventional echocardiographic parameters

**Figure 9:**
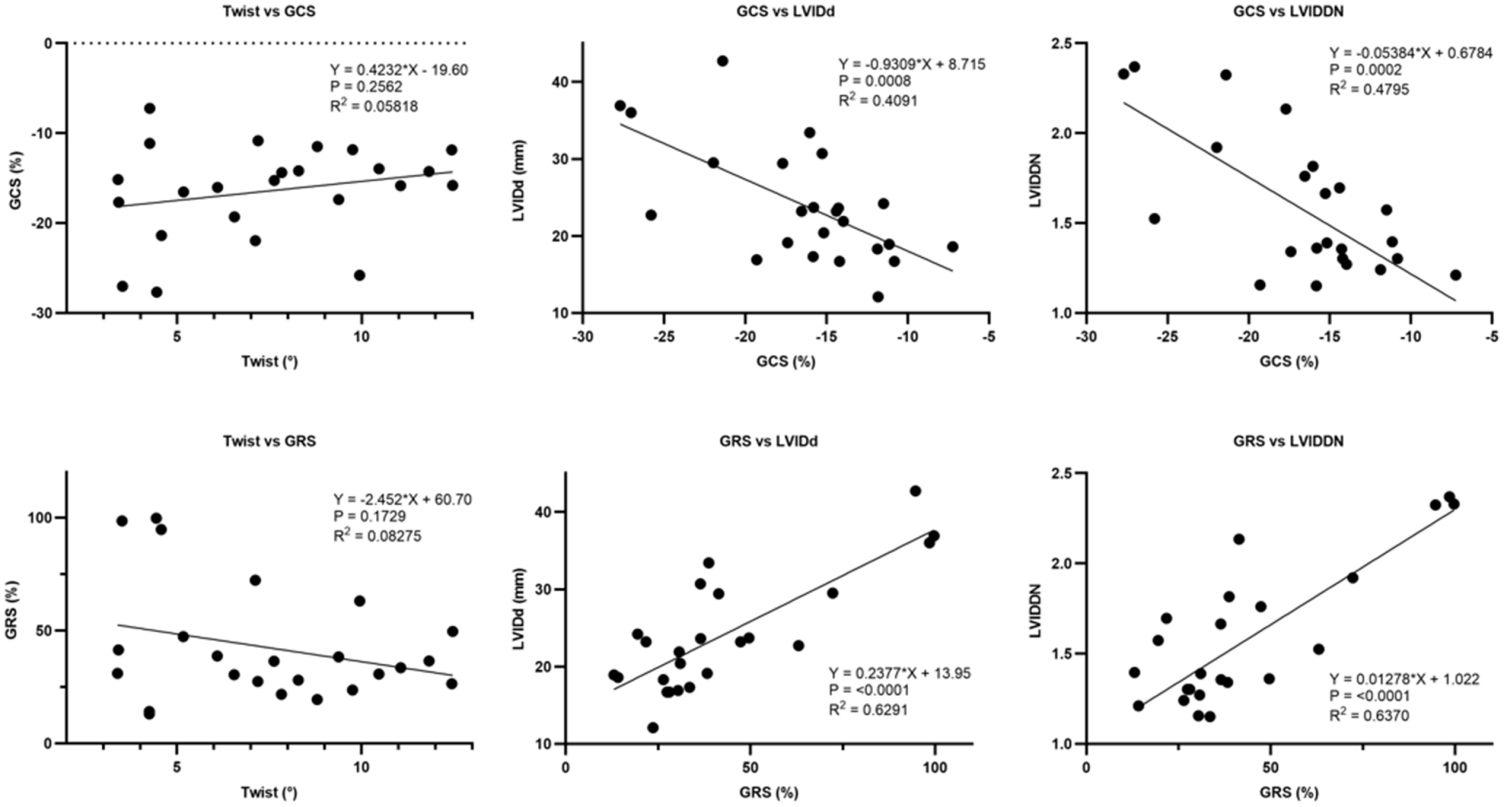
Linear regression analysis between global circumferential strain (GCS) and global radial strain (GRS) and twist, LVIDd, and LVIDDN. There was a significant effect of GCS on LVIDd and LVIDDN. Similarly, GRS exhibited a significant effect on LVIDd and LVIDDN. However, there was no significant effect of GCS or GRS on twist.

The coefficient of determination (*R*^2^) obtained from linear regression analysis between global circumferential strain (GCS) or global radial strain (GRS) and twist, LVIDd, and LVIDDN was illustrated in Figure 9 twist was not affected by GCS and GRS. However, GRS was significantly and moderately affected by LVIDd (*R*^2^ = 0.629, *p* = 0.0001) and LVIDDN (*R*^2^ = 0.637, *p* = 0.0001). Furthermore, there was negative effect of LVIDd (*R*^2^ = 0.409, *p* = 0.0008) and LVIDDN (*R*^2^ = 0.479, *p* = 0.0002) on circumferential strain.

### 3.4 Effect of 2D-STE parameters obtained from LV on RVFW-GLS

**Figure 10:**
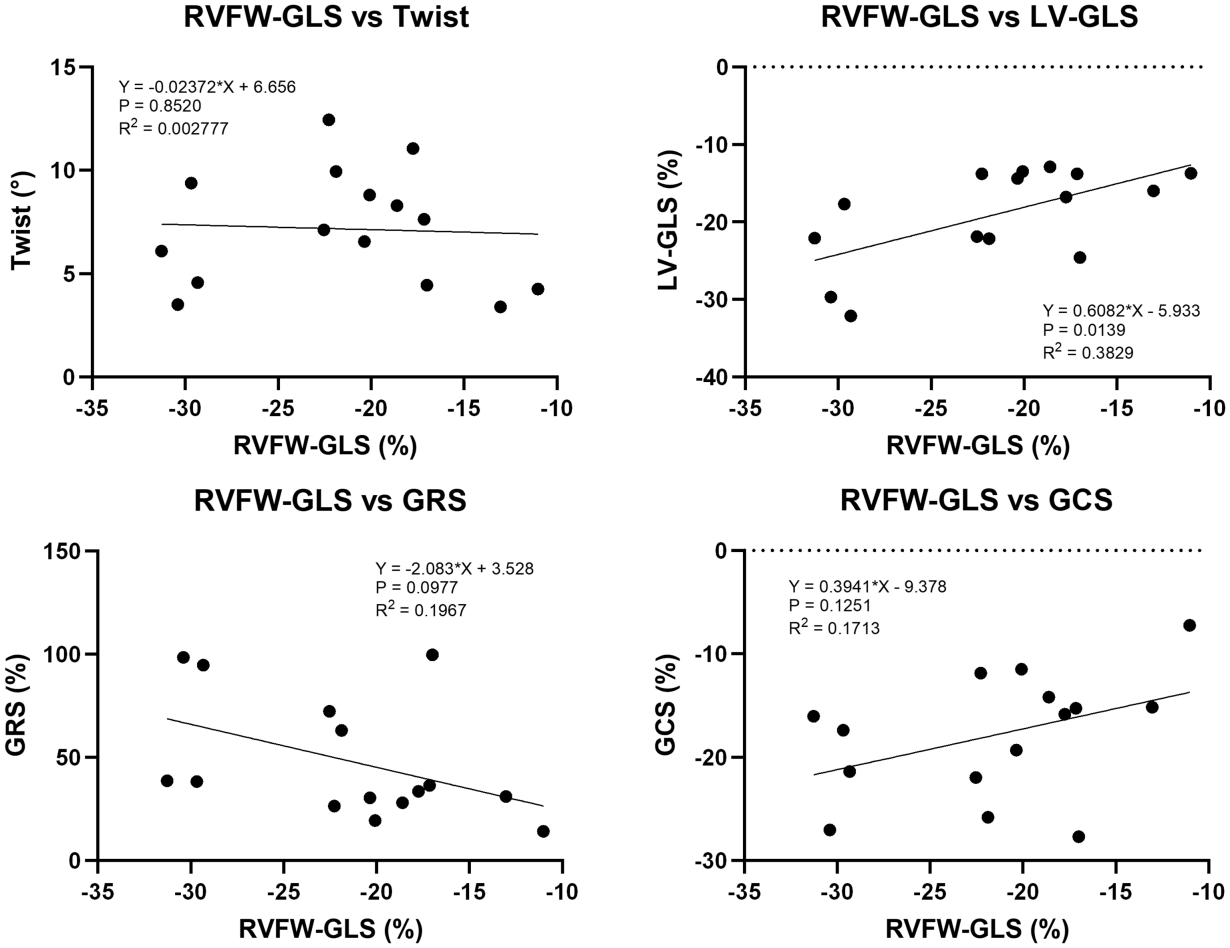
Linear regression analysis between 2D-STE parameters obtained from LV and RVFW-GLS. LV-GLS had a mild significant effect on RVFW-GLS. On the other hand, there was no significant effect of RVFW-GLS on twist, GCS and GRS.

The coefficient of determination (*R*^2^) obtained from linear regression analysis between twist, LV-GLS, GCS, GRS, and twist was illustrated in Figure 10. RVFW-GLS was not affected by twist, GCS, and GRS. The collective data showed a mild significant effect of LV-GLS on RV-GLS (*R*^2^ = 0.3829, *p* = 0.0139).

## 4 Discussion

Through the implementation of MVP, although twist did not reach the standard value, it showed an improvement trend, and GCS and GRS improved to near the standard value. Furthermore, at 3 months after surgery, it was suggested that there was an increase in left ventricular load and subsequent reverse remodeling without the need for inotropic or diuretic drugs. These results underscore MVP’s potential as an independent intervention for managing cardiac conditions, offering promising long-term benefits over medical therapy.

In dogs classified as Class III according to the International Small Animal Cardiac Health Council (ISACHC) classification, previous studies have reported a significant decrease in twist compared to normal dogs and those classified as Class II (9). In our study, preoperative twist in severe cases classified as ACVIM Stage D was notably low. Although not statistically significant in severe cases of ACVIM Stage D, twist exhibited an increasing trend, suggesting that MVP may still offer promise as an effective treatment for severe cases. In contrast, reports in humans with advanced mitral regurgitation indicate less favorable postoperative outcomes linked to a decline in myocardial contractility (17). In our study, all cases showed favorable postoperative progress, possibly attributed to the infrequent occurrence in dogs of conditions where myocardial contractility decreases, as observed in humans with heart failure with reduced ejection fraction (HFrEF) (18). Additionally, the absence of significant improvement in twist at 1 month postoperatively likely indicates that early postoperative myocardial damage or stress, potentially caused by surgical intervention or cardiopulmonary bypass, had not yet fully resolved. Over time, however, as the myocardium healed and recovered from the surgical trauma, twist values began to improve, as seen at the 3-month postoperative check-up. This gradual recovery process is consistent with past findings that it takes time for the heart muscle to recover after heart surgery (19).

Furthermore, reports from human medicine indicate a deterioration in postoperative left ventricular global longitudinal strain (LV-GLS) in patients with isolated primary mitral regurgitation (20), this trend was also observed in our study, as LV-GLS fell below the reference value after surgery. However, it is noteworthy that none of the cases in our study experienced worsened conditions postoperatively due to inadequate cardiac output, suggesting the maintenance of cardiac output through mechanisms beyond longitudinal myocardial motion. This resilience may be attributed to the composition of myocardial fibers in normal canine myocardium, where the ratio of longitudinally oriented fibers to circumferentially arranged fibers is approximately 1:10 (21). Studies have indicated an increase in circumferential strain in dogs classified as Class II in the ISACHC classification compared to normal dogs and those classified as Class I (8). Therefore, movements of myocardial fibers in the circumferential direction, including torsional motion, may play a pivotal role in sustaining effective cardiac output.

Moreover, the presence of cases exhibiting low fractional shortening (FS) but high twist suggests that twist encompasses movements in the short-axis direction as well. Therefore, accurate evaluation of left ventricular function through twist analysis may provide a more comprehensive assessment of the need for postoperative cardiac medication.

In previous studies, right ventricular global longitudinal strain (GLS) has been reported to deteriorate after MVP (20, 22). However, in our study, although there was a decrease in right ventricular GLS post-surgery, these values did not indicate deterioration. This is because, in dogs with MMVD, tricuspid annular plane systolic excursion (TAPSE), which is an indicator of right ventricular contractility, shows a significant positive correlation with the size of the left atrium and left ventricle (23), and TAPSE/Ao, which is expressed as the ratio of TAPSE to aortic diameter, shows a positive correlation with LA/Ao (24), so it has been reported that right ventricular contraction is affected by the left heart system. In other words, it suggests that the excessive contraction of the right ventricle, attributed to the hypercontractile state induced by left ventricular load secondary to MMVD spreading through the interventricular septum, normalized following surgery due to the improvement in left ventricular load. This is consistent with reports that MVP reduces longitudinal contraction in the right ventricle (25, 26). Furthermore, reports in humans indicate a significant decrease in TAPSE and maximum systolic velocity of the tricuspid annulus (PSV) post-surgery, while there was no significant change in right ventricular ejection fraction (27). These findings suggest that vertical motion of the heart in the right ventricle may also be important. Additionally, the weak correlation observed between left ventricular GLS and right ventricular free wall GLS (RVFW-GLS) in our study underscores the influence of interventricular interaction mediated by the interventricular septum. These dynamics may involve short-axis contractions and bellows-like movements in the right ventricle (28), aspects not evaluated in our study.

Increased GCS and GRS in response to progressive left heart load due to MMVD have been documented (8). In this study, preoperative GCS and GRS were observed to be elevated, influenced by LVIDd and LVIDDN, suggesting an association with left ventricular volume overload. This phenomenon may reflect a compensatory mechanism of the myocardium to counter volume overload. Interestingly, postoperative GCS and GRS improved to levels comparable to those reported in healthy dogs (15), indicating that MVP effectively alleviates the burden on the left ventricular system without causing significant myocardial damage, despite the cardiac arrest induced by surgery. Furthermore, twist was not influenced by GCS and GRS, highlighting its distinct role as a mechanism of cardiac output in the left ventricle, separate from circumferential and short-axis myocardial motion. Our data indicates that LV twist is significantly influenced not only LVIDDN and LVIDd, but also by LVSI. Previous studies have reported that twist is influenced by cardiac morphology (29), and our findings suggest that effective reverse remodeling of the left ventricular system following MVP can enhance left ventricular twisting motion, potentially contributing to motion restoration. Additionally, it has been reported that LVSI decreases with the progression of MMVD (30), suggesting that postoperative improvement in preload may alleviate excessively increased wall stresses as the left ventricular morphology returns to normal.

Previous reports in human medicine have shown variability in postoperative twist changes, with some groups experiencing increased left ventricular end-diastolic volume (LVEDV) and others showing a decrease (31). Similarly, it is conceivable that the degree of recovery in dogs may vary by stage as well. This suggests that postoperative outcomes may differ depending on the extent of preoperative myocardial impairment. In our study, some Stage B2 patients did not exhibit significant changes in twist between the preoperative and postoperative periods, suggesting that patients at lower stages may experience a milder decline in left heart function. However, the maintenance or recovery of twist in all patients despite discontinuation of inotropic drugs compared to preoperative levels suggests that MVP may be an effective treatment across all stages.

## 5 Limitations

One of the limitations of this study is that there were no healthy individuals or Stage B2 cases before or after treatment with pimobendan to use as a control group, so the treatment effect was judged using the reference values from previous reports. The reference values used in this study may not necessarily be appropriate, as they may vary depending on the size and breed of the dog. It is also known that the results can vary depending on the echo device used and the analysis software (32).

For this reason, it may be important to focus on the change in values before and after MVP.

In addition, the small number of cases in each stage means that it may not be possible to draw strong conclusions about the evaluation of cardiac function after surgery based on this study alone. However, it has become statistically clear that the therapeutic effect of MVP manifests in the same way in all cases.

This is thought to be a result that strongly supports the idea that MVP is one of the beneficial treatments, even with a small sample size.

Furthermore, in Stage B2, there were cases where diuretics were used in the absence of heart failure, and it is unclear whether the case was Stage B2 or Stage C.

In addition, as this study is a short-term follow-up survey of up to 3 months after surgery, it is impossible to deny the possibility that the trend of improvement in various parameters will disappear if the patient’s condition is followed over a long period of time.

## 6 Conclusion

Our study highlights 2D-STE analysis as a valuable tool for assessing the efficacy of MVP in treating MMVD, showing improvements in myocardial function. According to our knowledge, our study presents the first report of postoperative enhancement in twist, GCS, and GRS with a notable trend towards improvement even in severe cases, although statistical significance was not achieved. Moreover, our findings indicate that changes in the ventricular septum influence both cardiac systems. These results collectively underscore the promising potential of MVP in improving cardiac function and warrant further investigation into its clinical applicability and long-term benefits for MMVD patients.

## Data Availability

The original contributions presented in the study are included in the article/supplementary material, further inquiries can be directed to the corresponding authors.
